# Secreted Gaussia Luciferase as a Biomarker for Monitoring Tumor Progression and Treatment Response of Systemic Metastases

**DOI:** 10.1371/journal.pone.0008316

**Published:** 2009-12-15

**Authors:** Euiheon Chung, Hiroshi Yamashita, Patrick Au, Bakhos A. Tannous, Dai Fukumura, Rakesh K. Jain

**Affiliations:** 1 Edwin L. Steele Laboratory, Department of Radiation Oncology, Massachusetts General Hospital, Harvard Medical School, Boston, Massachusetts, United States of America; 2 Molecular Neurogenetics Unit, Department of Neurology, Massachusetts General Hospital, Harvard Medical School, Boston, Massachusetts, United States of America; 3 Department of Radiology, Center for Molecular Imaging Research, Massachusetts General Hospital, Harvard Medical School, Boston, Massachusetts, United States of America; City of Hope National Medical Center, United States of America

## Abstract

**Background:**

Currently, only few techniques are available for quantifying systemic metastases in preclinical model. Thus techniques that can sensitively detect metastatic colonization and assess treatment response in real-time are urgently needed. To this end, we engineered tumor cells to express a naturally secreted Gaussia luciferase (Gluc), and investigated its use as a circulating biomarker for monitoring viable metastatic or primary tumor growth and their treatment responses.

**Methodology/Principal Findings:**

We first developed orthotopic primary and metastatic breast tumors with derivative of MDA-MB-231 cells expressing Gluc. We then correlated tumor burden with Gluc activity in the blood and urine along with bioluminescent imaging (BLI). Second, we utilized blood Gluc assay to monitor treatment response to lapatinib in an experimental model of systemic metastasis. We observed good correlation between the primary tumor volume and Gluc concentration in blood (R^2^ = 0.84) and urine (R^2^ = 0.55) in the breast tumor model. The correlation deviated as a primary tumor grew due to a reduction in viable tumor fraction. This was also supported by our mathematical models for tumor growth to compare the total and viable tumor burden in our model. In the experimental metastasis model, we found numerous brain metastases as well as systemic metastases including bone and lungs. Importantly, blood Gluc assay revealed early growth of metastatic tumors before BLI could visualize their presence. Using secreted Gluc, we localized systemic metastases by BLI and quantitatively monitored the total viable metastatic tumor burden by blood Gluc assay during the course of treatment with lapatinib, a dual tyrosine kinase inhibitor of EGFR and HER2.

**Conclusion/Significance:**

We demonstrated secreted Gluc assay accurately reflects the amount of viable cancer cells in primary and metastatic tumors. Blood Gluc activity not only tracks metastatic tumor progression but also serves as a longitudinal biomarker for tumor response to treatments.

## Introduction

The evaluation of the metastatic tumor burden is complicated. Oftentimes, it can only be assessed at the sacrificial end point and longitudinal information on the progression remains unknown. This is especially problematic for evaluating treatments since tumor size at the start of treatment can vary considerably. Bioluminescence imaging (BLI) is a powerful tool for localizing and quantifying metastatic tumor growth. However, the spatial resolution of BLI is relatively poor and the optical signal propagation through living tissue compromises sensitivity and complicates accurate measurements, thus rendering the evaluation of small metastatic cell clusters rather difficult, if not impossible [1]. Secreted reporters in the blood have emerged as promising tools for the detection, quantification and noninvasive monitoring of biological processes in experimental models [2], [3], [4], [5], [6], [7], [8], [9]. Recently, naturally secreted Gaussia luciferase (Gluc) from the marine copepod *Gaussia princeps* has been demonstrated to be a sensitive and quantitative method for evaluating cancer cells *in vivo* [2]. Gluc has several advantages over other commonly used reporters for *in vivo* imaging. Gluc is 2000-fold more sensitive than firefly or *Renilla* luciferases and 20,000-fold more sensitive than the secreted alkaline phosphatase [4], [10]. Further, since Gluc is secreted, its concentration in the blood correlates with expression level in a given biological system [2], [11]. Here, we seek to evaluate secreted Gaussia luciferase as a novel biomarker for longitudinal monitoring of systemic metastasis.

We engineered MDA-MB-231BR (231BR) cells, a subline of human breast adenocarcinoma cell line (MDA-MB-231) selected from brain metastasis, to express Gluc in an experimental metastasis model [12]. In this model numerous brain metastases as well as systemic metastases including bone or lungs are observed. We utilized secreted Gluc to track the progression of 231BR cells that metastasized to various organs. To monitor treatment response by secreted Gluc assay, we treated mice with lapatinib, a dual kinase inhibitor that targets EGFR and Her2 [13]. Lapatinib was shown previously to reduce the outgrowth of brain tumors of MDA-MB-231BR-Her2 (231BR-Her2) [14]. Here we successfully demonstrate secreted Gluc as a new measure of viable tumor burden in primary and metastatic tumor models. We also show for the first time that blood Gluc assay allows the monitoring of treatment response in a metastasis model by synchronizing the treatment initiation with Gluc-matched tumor burden, a parameter typically difficult to determine. Our reported method will facilitate the study of the biology and treatment of metastatic disease using animal models.

## Results and Discussion

### Monitoring Orthotopic Primary Tumor Progression by Gluc Activity in Blood and Urine

**Figure 1:**
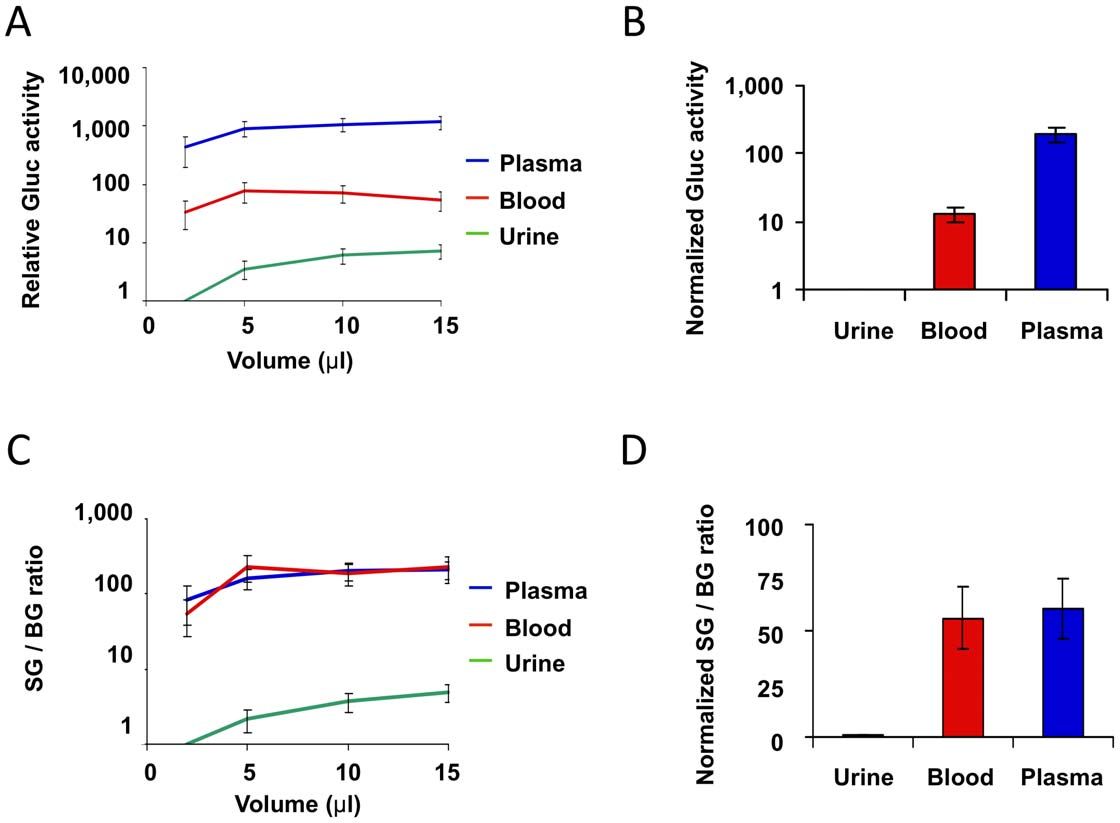
Comparison of Gluc activity in whole blood, plasma, and urine. 231BR-G tumor fragments were implanted in mammary fat pad. Blood and urine samples were collected from tumor-bearing animals and Gluc activity was evaluated in different sample volumes. **A**, The relative Gluc signal is plotted against different volume of each sample. Results shown are from three different tumor-bearing animals. Relative Gluc signal is obtained based on the corresponding 2 µl urine Gluc signal. **B**, The Gluc values from 10 µL samples normalized to urine Gluc values are plotted. **C**, The Signal-to-background ratio (SG/BG) was plotted against sample volume. The background is defined as the signal obtained from samples collected from control mice without tumors. Results are normalized by the corresponding 2 µl urine SG/BG. **D**, SG/BG ratio of the 10 µL volume of samples normalized to urine Gluc signals are plotted. All results were presented as mean ± SE (n = 3).

To correlate total primary tumor burden with Gluc activity, we first implanted 231BR-G cells orthotopically in the mammary fat pad to grow as a primary tumor. We compared the Gluc assay signals in the plasma, whole blood, and urine. Gluc signals were highest in plasma, followed by blood, and urine that exhibited the lowest signal (Fig. 1*A, B*). Even though Gluc in plasma gave a higher signal, the signal-to-background ratio (SG/BG ratio) of Gluc in blood was comparable to that of Gluc in plasma, and both were higher than that of the urine by two orders of magnitude (Fig. 1*C, D*). Since additional centrifugation step and twice more volume of blood are required for plasma collection, we used blood Gluc for our subsequent study.

**Figure 2:**
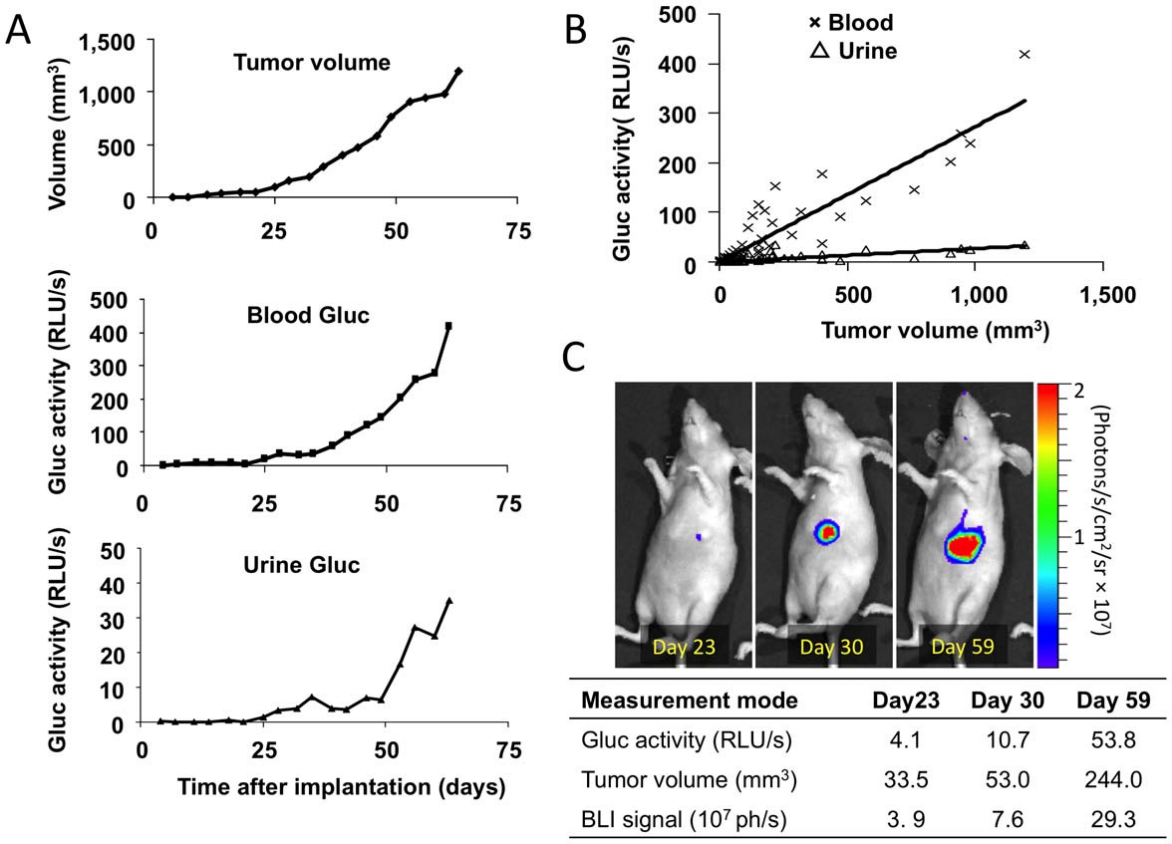
Monitoring of Gluc activity in blood, urine, and bioluminescence imaging (BLI) of orthotopic breast cancer model. 231BR-G tumor fragments were implanted in the mammary fat pad. **A–C**, Blood and urine were collected twice a week from 4 to 63 days after implantation and Gluc activity was acquired using a luminometer (n = 3; 10 µl of blood and 5 µl of urine). At the same time, tumor volume was quantified by caliper measurements. **A**, Representative time-course data of tumor volume, blood and urine Gluc activity in one representative animal, **B**, Correlation of the blood or urine Gluc value to the tumor volume, **C**, Representative bioluminescent images with concurrent measurement values of blood Gluc value, tumor volume, and BLI signal at days 23, 30, and 59 after tumor implantation.

Primary tumor growth was assessed with three different modalities - Gluc activity, tumor volume estimation, and BLI signal. The blood Gluc value correlated well (R^2^ = 0.84) with tumor volume. Urine Gluc value also correlated with the tumor volume but to a lesser extent (R^2^ = 0.55) (Fig. 2*A, B*). We also confirmed that primary tumors detected with blood Gluc assay could also be detected with whole body BLI (Fig. 2*C*).

### Whole Blood Gluc Activity Reflects Viable Tumor Volume

**Figure 3:**
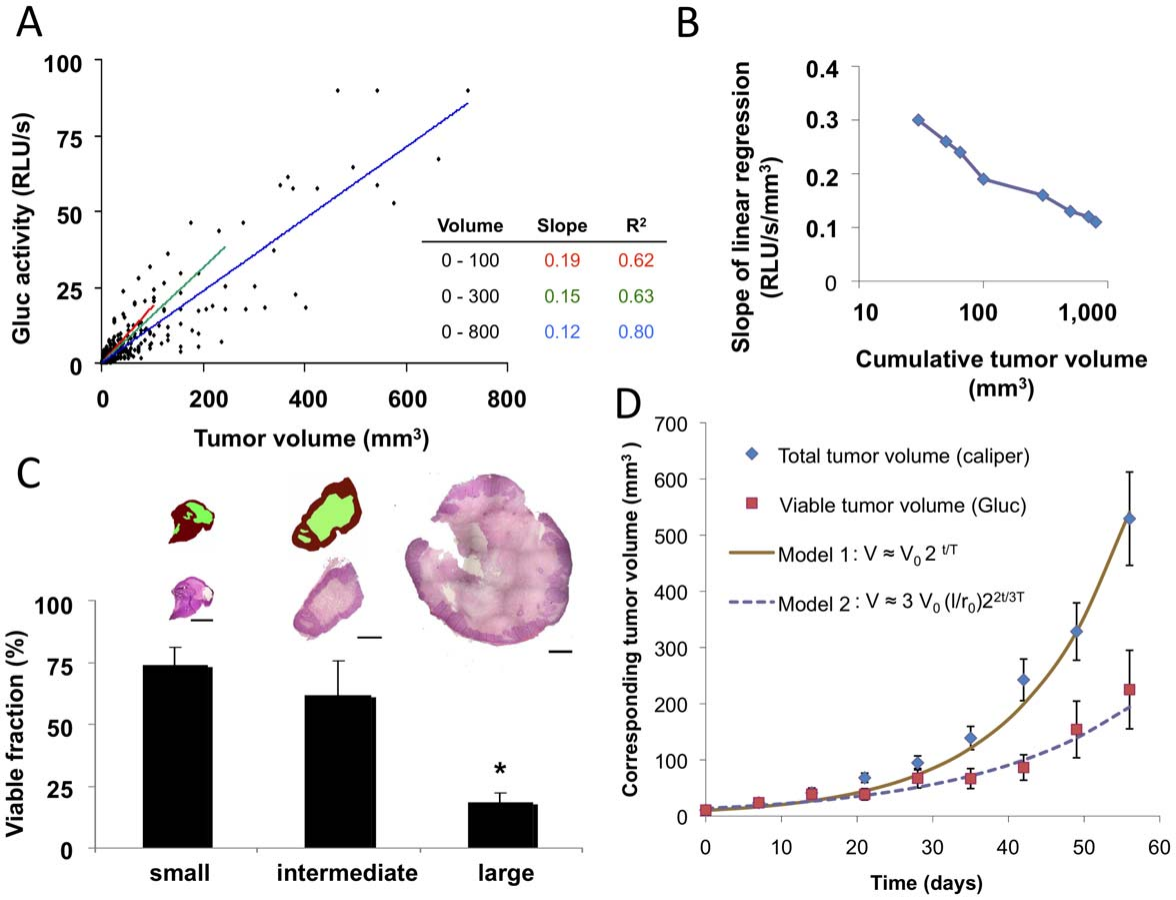
Blood Gluc assay reflects viable tumor burden. 231BR-G tumor fragments were implanted in the mammary fat pad. Blood Gluc and tumor volume were assessed twice a week (n = 24). **A**, Scatter plot of blood Gluc values and tumor volumes up to 9 weeks after the implantation. Linear regression was analyzed for three tumor volume ranges and specified with slopes and R^2^ values. **B**, Slope of the line of linear regression line with respect to the cumulative tumor volume range. **C**, Viable tumor fraction analyzed using H&E stains of central tumor sections. Final tumor sizes are segregated into three groups and viable fraction is shown as mean ± SE (n = 3–5 tumors per group, small: 0–100, intermediate: 100–300, large: 300–800 mm^3^). *, P < 0.05 by Mann-Whitney U-test when small and intermediate tumors are compared to large tumors. Two illustrative figures with viable tumor segregation are shown (red: viable tumor, green: necrotic area). Scale bar is 1 mm. **D**, Mathematical modeling to fit the measured total tumor volume and viable tumor volume estimated from blood Gluc activity. Model 1 fits total tumor growth and Model 2 fits viable tumor growth.

It is not clear whether blood Gluc value accurately represents tumor volume. To investigate their relationship closely, we analyzed blood Gluc value and primary tumor volume over 9 weeks. Overall, blood Gluc activity correlates with tumor volume well (Fig. 3*A*). Interestingly, we observed that the slope of the line of linear regression decreases as the range of cumulative tumor volume increased (Fig. 3*B*). After grouping the tumors in three different volume ranges (0–100, 0–300, 0–800 mm^3^), the respective linear regression line shows a consistent decrease in the slope as the group includes bigger tumors (Fig. 3*A* and inset table, and Fig. 3*B*). We hypothesized that this phenomenon may be in part due to an increasing fraction of necrotic tissue in the bigger tumor (Fig. 3*C* and Methods). Since Gluc is secreted by viable tumor cells, the blood Gluc signal would reflect total viable tumor volume while size-measurement represents total tumor volume including necrotic tissue. To understand this mechanistically, we developed a simple mathematical model to explain the observed tumor growth data of total tumor volume and viable tumor volume with blood Gluc value (Fig. 3*D*, Table S2). We assumed i) blood Gluc signal is proportional to the volume of viable tumor cells, ii) tumor volume is proportional to the number of total tumor cells, and iii) the viable tumor rim thickness is constant with central tumor necrosis. To convert the blood Gluc activity into the corresponding tumor volume, we normalized each blood Gluc value - by dividing Gluc value at day 0 and by multiplying the corresponding tumor volume at day 0 (defined by tumor volume of ∼10 mm^3^). The curve fittings were made by two spherical tumor growth models: Model 1 is based on exponential tumor growth curve for tumor volume measurement, and Model 2 is based on central necrosis and a viable tumor rim. The fitting of Model 2 with corresponding tumor volume from Gluc measurements provides the viable tumor rim of 0.6 mm that agrees with the viable tumor rim observed in the hematoxylin & eosin (H&E) analyses in Fig. 3*C* (Details in Supporting Information S1). These data support that blood Gluc activity reflects viable tumor burden. In addition, blood Gluc assay potentially provides more precise measurement since manual tumor volume measurement is inherently operator dependent.

### Real-Time Monitoring of Metastatic Tumor Progression with Blood Gluc and Localization of Metastases with Gluc Bioluminescence Imaging

**Figure 4:**
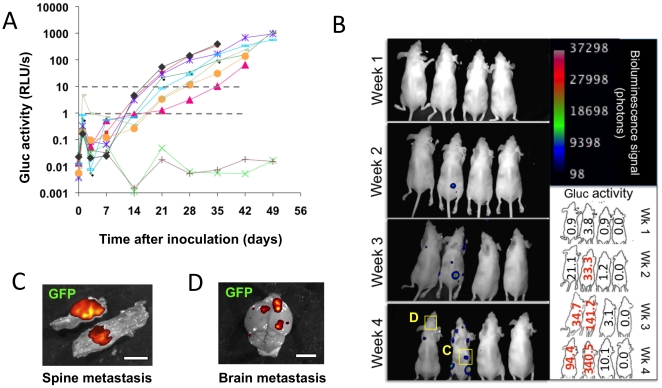
Real-time monitoring of metastatic tumor progression with blood Gluc assay and localization of metastases with bioluminescence imaging. **A**, 231BR-Her2-G cells were injected in the heart (n = 10). Blood Gluc was measured at day 0 (before injection), 1, 3 days, and weekly. **B**, Longitudinal BLI with concurrent blood Gluc values (four representative animals are shown). Each Gluc value is shown in the right lower inset. Bold red values are the animals whose metastases are detectable in the BLI. **C–D**, *Ex vivo* images of spine (C) and brain (D) metastatic tumors overlayed with GFP signals taken with the same IVIS system. Scale bar is 5 mm.

We hypothesized that blood Gluc assay provides a sensitive measurement for monitoring of systemic metastasis and BLI with Gluc shows localization of metastasis. To test this hypothesis, we utilized an experimental metastasis model by injecting 231BR-Her2-G cells into the arterial circulation. We observed that blood Gluc signal sharply increased one day after cell injection and then, the signal dropped precipitously to the basal level three days after indicating massive loss of injected cells after initial short time survival (Fig. 4*A*). Blood Gluc value in 8 out of 10 mice reached 1 Relative Light Unit (RLU)/s at 14–21 days after inoculation. Interestingly, all animals with blood Gluc value above 1 RLU/s eventually developed systemic metastasis including bone and brain (Fig. 4*C, D*). We confirmed these data with 4 separate experiments. All 46 out of 77 mice with blood Gluc values greater than 1 RLU/s at 14–35 days after intra-cardiac injection, developed systemic metastasis (Table S1). In contrast, the 31 mice that did not have blood Gluc values above 1 failed to develop metastasis. Thus, blood Gluc assay allows early detection of systemic metastatic colonization and provides a means for quantitative evaluation of metastatic tumor burden in real-time. In most cases, we could not localize the metastatic sites with BLI when blood Gluc value was below 10 RLU/s (Fig. 4*B*). Big or superficial tumors were detectable with BLI (Fig. 4*B, C*) while small or deeply located tumors were not always recognizable (Fig. 4*B, D*). Even though BLI provides a powerful tool for longitudinal observations, the quantification of bioluminescent signal is limited due to the light scattering and absorption through the tissue [15], [16]. We next compared the BLI signal of Gluc with conventional Firefly luciferase (Fluc). We performed a control experiment using by Fluc and Gluc double-transfected cell line in the experimental metastasis model. Both showed comparable signal level and similar localization of metastasis (Fig. S1*B*, S1*C*). The peak emission wavelength of Gaussia luciferase is 480 nm [10], and therefore it has higher tissue absorption as compared to that of firefly luciferase with 612 nm at 35°C [17]. Despite this limitation, Gluc BLI is shown to be comparable to Fluc for imaging metastatic tumors due to its high photon flux. These results suggest that by engineering a non-secreted version of Gluc, this luciferase can potentially be more sensitive than Fluc in localizing metastasis *in vivo*. In fact, it has been shown recently that by either cloning an ER-retention signal or a transmembrane domain at the C-terminus of Gluc, that over 10-fold of this protein is retained in/on the cell leading to nearly one order of magnitude higher sensitivity in localizing cells *in vivo* as compared to the wild-type secreted Gluc [10], [18]. It should be noted that the 1RLU/s threshold value for detecting and initiating the treatment of metastasis was specific to the particular model system and luminometer setting used in this study. The threshold value is dependent on multiple variables including the sensitivity of a luminometer and levels of expression of Gaussia luciferase in a given cell line. In addition, the entry of Gluc into the circulation could be affected by the microenvironment and interaction between tumor and host cells.

### Blood Gluc Assay Provides Real-Time Monitoring of Treatment Response for Systemic Metastasis Progression

**Figure 5:**
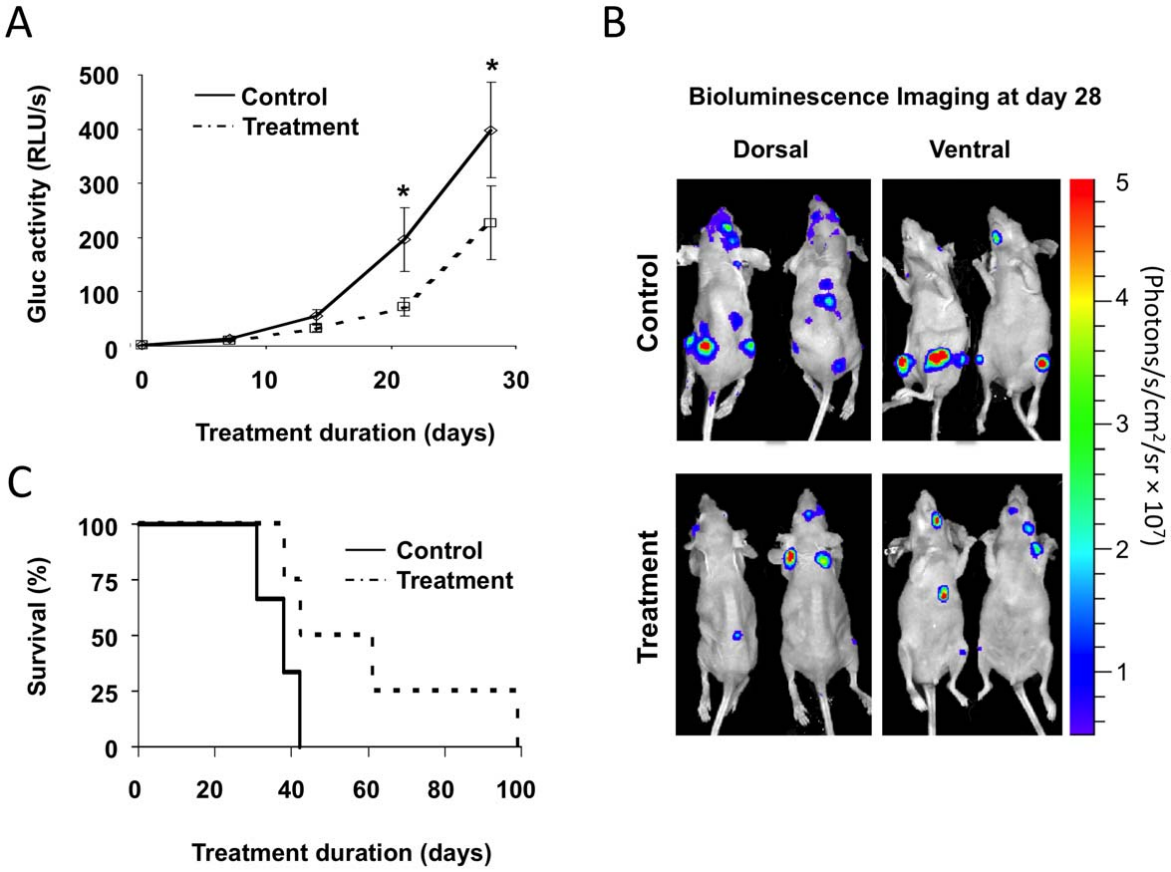
Blood Gluc assay provides real-time monitoring of treatment response for systemic metastasis progression. 231BR-Her2-G cells were injected into the heart. When blood Gluc value reached at 1 RLU/s, mice were treated with lapatinib. **A–B**, blood Gluc and BLI were assessed weekly (n = 16 per group). Mice were sacrificed 28 days after treatment for sample collection. *, P < 0.05 by Mann-Whitney U-test. Data shown as mean ± SE. **B**, BLI images of representative animals in each group (dorsal and ventral view) of at day 28 are shown. Imaging was done individually, **C**, Kaplan-Meier survival curve in lapatinib treated mice vs control animals (treatment  = 4, control  = 3).

Next we tested whether blood Gluc assay can be used to monitor treatment response in real-time with our metastasis model. We used the 231BR-Her2-G cells in the experimental metastasis model to examine whether lapatinib could inhibit systemic metastatic progression. When blood Gluc value reached at 1 RLU/s, mice were treated with lapatinib (100 mg/kg bid) or vehicle. Gluc value-matched starting points would minimize variations in initial metastatic tumor burden among treated animals which is inevitable in time-matched starting point [14]. With blood Gluc value, we showed that lapatinib suppressed the progression of systemic metastasis (Fig. 5*A*). Metastatic tumor progression was concurrently monitored by BLI imaging (representative BLI images in Fig. 5*B*). In control mice, we observed more extensive metastases compared to treated mice. We then investigated the effects of lapatinib treatment on mice survival by using Gluc-matched treatment initiation in Fig. 5*C*. All three mice in the control group did not survive beyond 42 days while two out of four treated mice survived much longer up to day 61 and 99, respectively. With the use of blood Gluc level, we showed that lapatinib significantly delayed the systemic tumor progression and prolonged survival in this model. Thus, blood Gluc activity can be used as a quantitative biomarker for longitudinal monitoring of tumor progression and treatment response in metastatic disease.

The field of metastasis research traditionally used Fluc as the reporter for bioluminescence imaging of tumor burden in vivo. The application of the Gluc reporter for blood assay will facilitate the detection of early systemic metastasis, particularly at a stage that is still not detectable by Fluc or Gluc-based bioluminescence imaging. Furthermore, the Gluc utilizes a different substrate (i.e. coelenterazine) than Fluc (i.e. luciferin) and this allows simultaneous monitoring of tumor burden and signaling pathway activity [19], [20]. In summary, we demonstrated that blood Gluc assay accurately measures the amount of viable cancer cells in primary and metastatic tumors. Blood Gluc assay is highly sensitive and it provides a novel way to longitudinally monitor metastatic progression and response to treatment. This technology will aid in the study of tumor metastasis and the development of strategies in treating this devastating disease.

## Materials and Methods

### Ethics Statement

All animal procedures were performed following the guidelines of Public Health Service Policy on Humane Care of Laboratory Animals and approved by the Institutional Animal Care and Use Committee of the Massachusetts General Hospital. Animals were anesthetized with ketamine/xylazine (100/10 mg/kg, intraperitoneal injection) for all experimental procedures.

### Cell Lines and Cell Culture

The 231BR cell line and its human epidermal growth factor receptor 2 (Her2)-overexpressing counterpart (231BR-Her2) both expressing green fluorescent protein (GFP) were kindly provided by Dr. Patricia S. Steeg (National Cancer Institute, Bethesda, MD) [21]. The 231BR and 231BR-Her2 cells were maintained in Dulbecco's modified Eagle Medium (DMEM, Mediatech, Manassas, VA) supplemented with 10% fetal bovine serum (FBS, Atlanta biologicals, Lawrenceville, GA) and 1% penicillin-streptomycin solution (SIGMA, St. Louise, MO).

### Gluc Transfection

The lentiviral vector carrying an expression cassette encoding Gluc and cerulean fluorescent protein (CFP, LV-Gluc-CFP) separated by an internal ribosomal entry site has been generated previously [2]. 231BR and 231BR-Her2 cells were transduced with LV-Gluc-CFP – 231BR-G and 231BR-Her2-G - at a multiplicity of infection of 50 as previously described [2]. CFP positive cells were sorted with a FACSAria cell sorter (BD Biosciences, San Jose, CA), at the Flow Cytometry Facility at the Ragon Insitute (Massachusetts General Hospital, Boston, MA).

### Orthotopic Breast Cancer Model (Mammary Fat Pad Model)

Tumor fragments from established 231BR-G tumors (volume of 200–300 mm^3^) were implanted in the mammary fat pad (MFP) of 5–7 weeks-old female nude mice. Tumor size was measured by a caliper. The following formula was used to calculate tumor volumes  =  (shorter diameter)^2^×(longer diameter)/2. Tumors were resected and used for H&E staining.

### Experimental Metastasis Model (Intracardiac Injection Model)

6–7 weeks old female nude mice were injected with 0.25×10^6^ 231BR-Her2-G cells in 0.1 ml PBS via the left ventricle [21]. Mice were euthanized when animals showing clinical symptom of prolonged distress or when they showed signs of neurological impairment or lost more than 20% of body weight defined as a survival end point. The brain, bone, and the other organs were harvested and immediately evaluated for GFP using fluorescent microscopy.

### Blood/Urine Gluc Assay

Measurement of secreted Gluc was performed as previously described [11]. Briefly, blood was drawn from making a slight nick in the tail-vein. Urine was collected directly from urethral openings. 10 µl of blood was collected and mixed with 2 µl of 50 mM EDTA. All the samples used for Gluc assay contain 20% (vol/vol) EDTA solution. Blood or urine sample was then transferred to a 96-well plate. Gluc activity was measured using a plate luminometer (MLX luminometer, Dynex technologies, Chantilly, VA). The luminometer was set to automatically inject 100 µl of 100 mM coelenterazine (CTZ, Nanolight, Pinetop, AZ) in PBS and photon counts were acquired for 10 sec.

### Viable Tumor Quantification

H&E stained section of the MFP tumors (one central cross section per tumor) were examined. Based on H&E staining, the region of viable tumor area in each tumor section was determined by a pathologist, blinded to the study. Most tumors had a predominantly necrotic core with a viable rim. The area of the viable tumor was quantified with respect to the full cross-sectional tumor area by a custom-written Matlab program. It is noteworthy that some breast tumors (e.g. MCaIV tumors) do not have clear viable tumor rim (unpublished observation). Since the mathematical model has not been tested in other tumor models, it warrants further examinations in the future.

### Treatment

Lapatinib was purchased from GlaxoSmithKline (Philadelphia, PA). Each lapatinib tablet was grounded and was dissolved in sterile water of 0.5% Tween80 (Sigma). Lapatinib treatment started when the whole blood Gluc value reached at 1 RLU/s. Lapatinib (100 mg/kg bodyweight) was administered twice a day by oral gavage.

### Bioluminescence Imaging

Individual animal was anesthetized and BLI was performed immediately after retro-orbital injection of CTZ (4 mg/kg body weight unless otherwise specified). IVIS Imaging System (Lumina II, Caliper Life Sciences, Hopkinton, MA) was used for BLI recording. The image acquisition time was in the range of 15 sec to 1 min. Post-processing and quantification was performed using Living Image software 3.0 coupled to the IVIS system. For BLI analysis of primary mammary tumors, photon flux was calculated for each mouse by using a circular region of interest encompassing the primary tumor in supine position.

### Statistical Analysis

Data were expressed as the mean ± SE. Statistical analysis was performed using two-sided Mann-Whitney U-test. Statistical significance was defined as P < 0.05. The survival curves were estimated by the Kaplan-Meier method.

## Supporting Information

Figure S1 Comparison of Fluc and Gluc for bioluminescence imaging of metastasis. MDA231BR cells were co-infected with 2 lentivirus vectors encoding Gluc-CFP and Fluc-mCherry. (A) Fluorescent microscopy images showing that these cells are equally expressing these reporters. Scale bar 50 µm. (B) MDA231BR cells expressing both Gluc and Fluc were inoculated via intracardiac injection into 7 weeks old female nude mice. Five weeks after inoculation, mice were imaged with either Gluc BLI after i.v. injection of coelenterazine (8 mg/kg body weight), or Fluc BLI after i.p. injection of D-luciferin (150 mg/kg body weight). Fluc BLI imaging was done at least 3 hrs after Gluc BLI imaging. (C) Quantification of Fluc and Gluc bioluminescent signals (photon flux) from 9 different metastatic regions in three animals.
(2.14 MB TIF)

Table S1 The blood Gluc values and the development of systemic metastasis. Once the blood Gluc value reaches over 1 RLU/s, all animals eventually developed detectable systemic metastasis with both blood Gluc and BLI. The time for reaching RLU/s of 1 varies among animals from day 14 to 35 after intracardiac injection of the MDA231BR-G cells. The results are from four separate experiments.
(0.35 MB DOC)

Table S2 Experimental data of orthotopic tumor growth with caliper and Gluc blood measurements. Size-matched tumor growth data of both total tumor volume and viable tumor volume with MDA-MB-231BR tumors-expressing Gluc.(All volumes are in mm^3^, n = 11)
(0.03 MB DOC)

Suppporting Information S1 Mathematical modeling of total tumor and the viable tumor volume. A mathematical modeling approach to correlate the caliper measurement to the total tumor volume, and to correlate the blood Gluc assay to the viable tumor burden.
(0.08 MB DOC)
