# Supplementary figures and images for: Secreted Gaussia Luciferase as a Biomarker for Monitoring Tumor Progression and Treatment Response of Systemic Metastases
Source: PLoS One. 2009 Dec 15;4(12):e8316. doi: 10.1371/journal.pone.0008316 (PMC2789383; doi:10.1371/journal.pone.0008316)

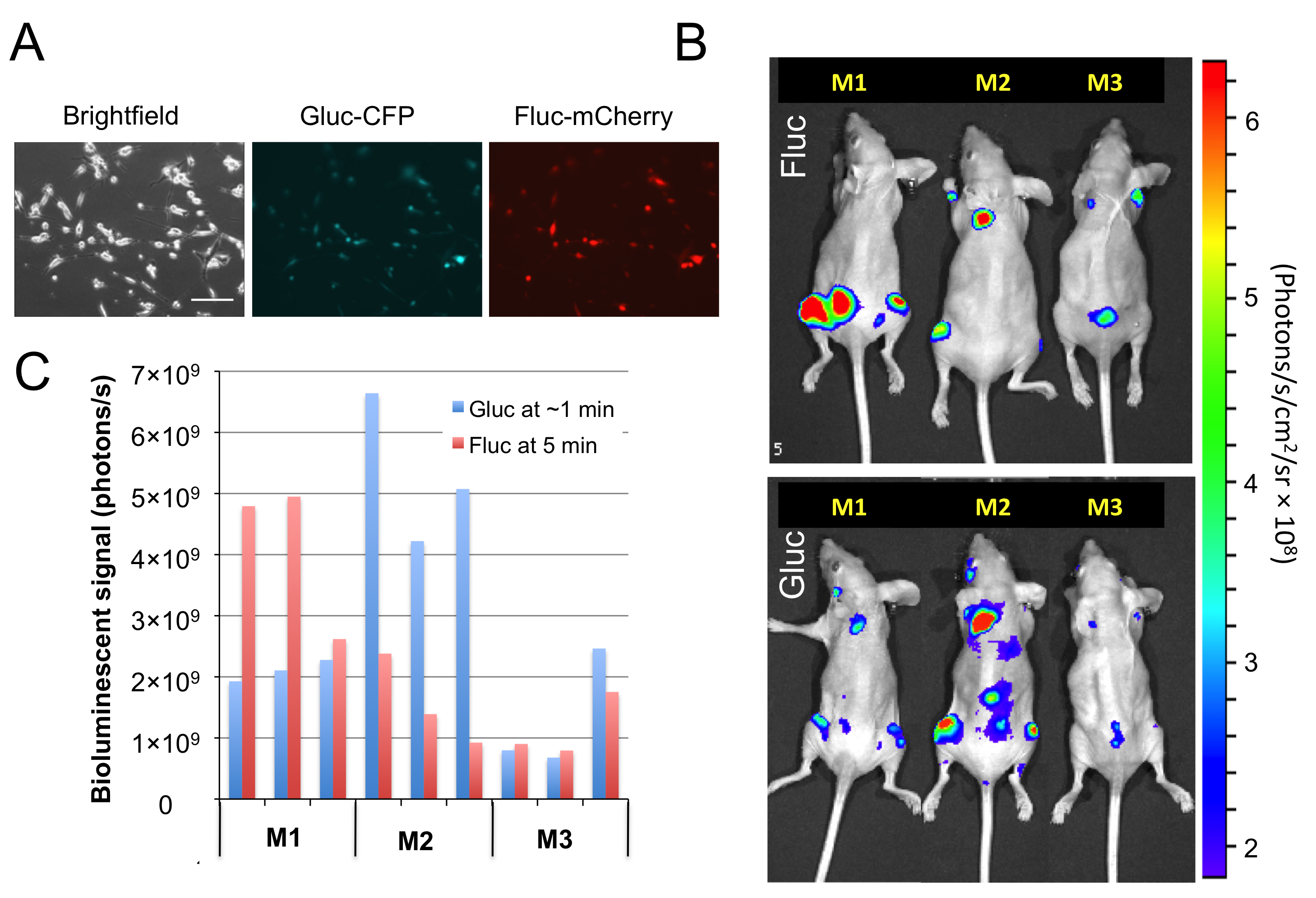

Supplement: Figure S1 — Comparison of Fluc and Gluc for bioluminescence imaging of metastasis. MDA231BR cells were co-infected with 2 lentivirus vectors encoding Gluc-CFP and Fluc-mCherry. (A) Fluorescent microscopy images showing that these cells are equally expressing these reporters. Scale bar 50 µm. (B) MDA231BR cells expressing both Gluc and Fluc were inoculated via intracardiac injection into 7 weeks old female nude mice. Five weeks after inoculation, mice were imaged with either Gluc BLI after i.v. injection of coelenterazine (8 mg/kg body weight), or Fluc BLI after i.p. injection of D-luciferin (150 mg/kg body weight). Fluc BLI imaging was done at least 3 hrs after Gluc BLI imaging. (C) Quantification of Fluc and Gluc bioluminescent signals (photon flux) from 9 different metastatic regions in three animals. (2.14 MB TIF) [file pone.0008316.s001.tif]

**
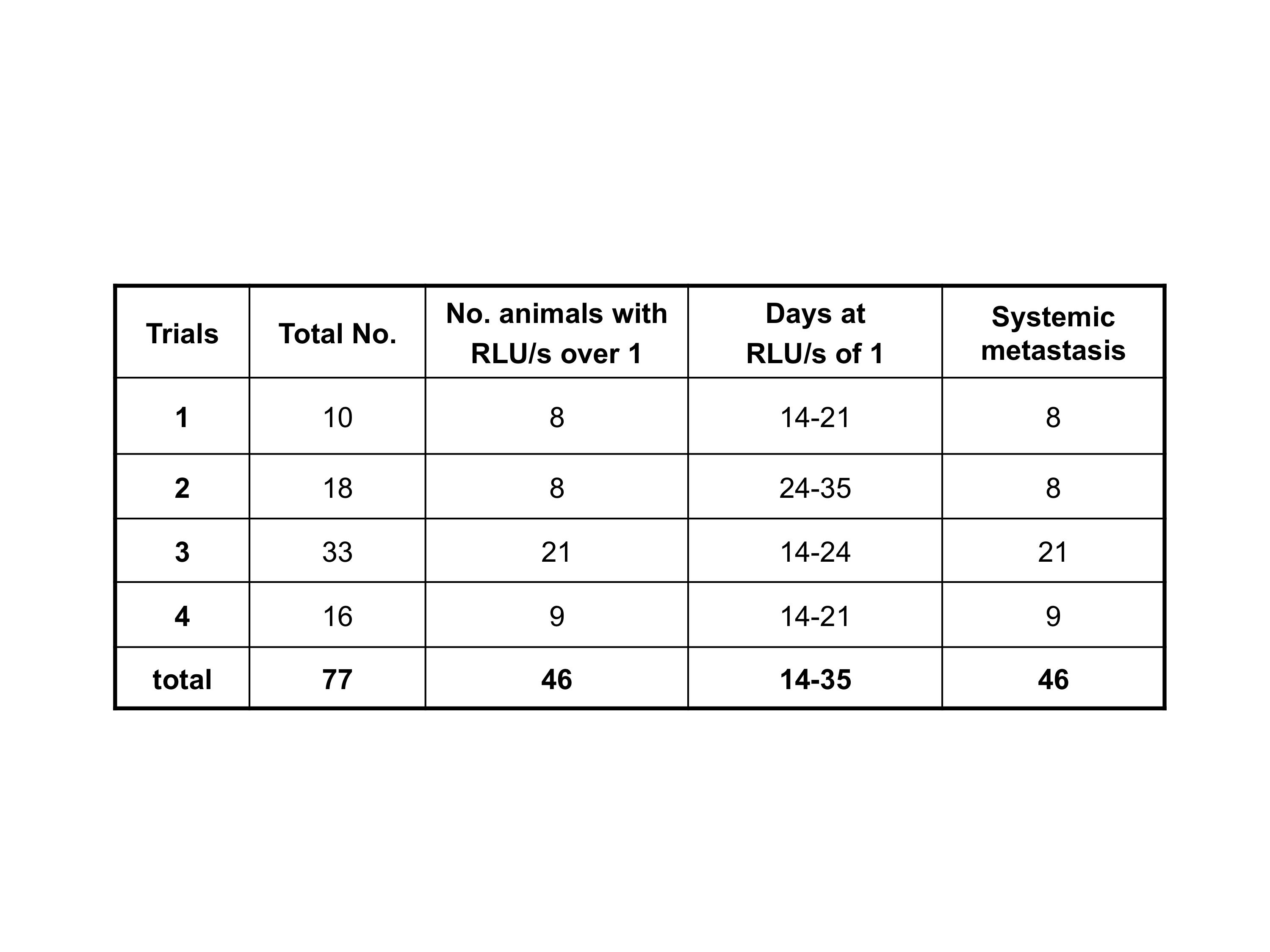
**

Supplement: Table S1 — The blood Gluc values and the development of systemic metastasis. Once the blood Gluc value reaches over 1 RLU/s, all animals eventually developed detectable systemic metastasis with both blood Gluc and BLI. The time for reaching RLU/s of 1 varies among animals from day 14 to 35 after intracardiac injection of the MDA231BR-G cells. The results are from four separate experiments. (0.35 MB DOC) [file pone.0008316.s002.doc]
