# Supplementary material for: Secreted Gaussia Luciferase as a Biomarker for Monitoring Tumor Progression and Treatment Response of Systemic Metastases
Source: PLoS One. 2009 Dec 15;4(12):e8316. doi: 10.1371/journal.pone.0008316 (PMC2789383; doi:10.1371/journal.pone.0008316)
Supplement: Table S2 — Experimental data of orthotopic tumor growth with caliper and Gluc blood measurements. Size-matched tumor growth data of both total tumor volume and viable tumor volume with MDA-MB-231BR tumors-expressing Gluc.(All volumes are in mm3, n = 11) (0.03 MB DOC) [file pone.0008316.s003.doc]

| **Date** | **day 0** | **day 7** | **day 14** | **day 21** | **day 28** | **day 35** | **day 42** | **day 49** | **day 56** |
| --- | --- | --- | --- | --- | --- | --- | --- | --- | --- |
| **Total tumor (caliper)** | 10.7 | 23.7 | 41.4 | 68.1 | 94.7 | 138.9 | 242.5 | 328.4 | 529.3 |
| **Viable tumor (Gluc)** | 10.7 | 23.8 | 39.1 | 38.7 | 67.4 | 66.7 | 86.5 | 154.2 | 225.2 |
| **SE(caliper)** | 0.8 | 2.2 | 5.7 | 8.3 | 12.4 | 20.7 | 37.0 | 51.1 | 83.1 |
| **SE(Gluc)** | 1.3 | 5.7 | 10.8 | 10.1 | 17.3 | 17.8 | 22.6 | 50.4 | 69.9 |
