# Supplementary material for: Secreted Gaussia Luciferase as a Biomarker for Monitoring Tumor Progression and Treatment Response of Systemic Metastases
Source: PLoS One. 2009 Dec 15;4(12):e8316. doi: 10.1371/journal.pone.0008316 (PMC2789383; doi:10.1371/journal.pone.0008316)
Supplement: Suppporting Information S1 — Mathematical modeling of total tumor and the viable tumor volume. A mathematical modeling approach to correlate the caliper measurement to the total tumor volume, and to correlate the blood Gluc assay to the viable tumor burden. (0.08 MB DOC) [file pone.0008316.s004.doc]

**Supporting Information:**

**Mathematical modeling of total tumor and the viable tumor volume**

We describe a mathematical modeling approach to correlate the caliper measurement to the total tumor volume, and to correlate the blood Gluc assay to the viable tumor burden. The models proposed here are based on the following three simplifying assumptions: 1) Tumor has a spherical shape; 2) The signal from blood Gluc assay is proportional to the volume of viable tumor cells; and 3) With central tumor necrosis, the viable tumor rim has a constant thickness.

**Model 1: A mathematical model of total tumor growth to fit caliper-based tumor volume measurement**

The initial tumor volume with initial radius $r_{0}$ can be written as $V_{0}=\left(4\pi/3\right)r_{0}^{3}$.

The tumor volume $V_{1}$ with radius $r$ can be described as:

(S.1)

where $T$is the tumor doubling time and is the normalized tumor radius. By fitting this equation to the tumor volume measurement, the tumor doubling time $T$can be obtained.

**Model 2: A mathematical model of viable tumor growth with central necrosis to fit blood Gluc tumor volume estimation**

This tumor model is to estimate the viable tumor burden assuming the existence of central tumor necrosis while the viable tumor thickness at the rim of the tumor remains constant. The viable tumor volume $V_{2}$ with outer radius $r$ and viable tumor rim thickness of $l$ can be described as follows.

$\begin{matrix} V_{2}=\frac{4\pi}{3}\left[r^{3}-\left(r-l\right)^{3}\right]=\frac{4\pi}{3}l\left(3r^{2}-3lr+l^{2}\right) \\ \approx\frac{4\pi}{3}l\left(3r^{2}\right)=3\left(\frac{l}{r_{0}}\right)V_{0}\left(\frac{r}{r_{0}}\right)^{2}=3\left(\frac{l}{r_{0}}\right)V_{0}\cdot2^{\frac{2t}{3T}} \end{matrix}$ (S.2)

This formulation only holds for the condition when$r$is bigger than $l$, which is true in most cases. By fitting the equation (S.2) to the corresponding tumor volume by the blood Gluc value using the tumor doubling time $T$(obtained from Model 1), the viable tumor rim $l$ can be estimated.

We applied these two Models to fit the data of primary tumor growth with both total tumor and viable tumor volume. Total tumor volume is calculated by caliper measurement and viable tumor volume is estimated by the blood Gluc values (i.e. normalized Gluc value by its value at day 0 and multiplied by the total tumor volume at day 0, $\left(G/G_{0}\right)V_{0}$). Supplementary Table S2 shows the size-matched tumor growth data of both total tumor volume and viable tumor volume obtained with 11 animals bearing MDA-MB-231BR tumors-expressing Gluc growing at the mammary fat pad.

By fitting the total tumor volume data to Model 1, we can estimate the overall tumor doubling time $T$ = 9.7 days with R^2^ = 0.97. Furthermore, fitting the viable tumor volume to Model 2 using the same tumor doubling time, we can estimate that the viable tumor rim is 0.60 mm with R^2^ = 0.97. Fig. 3*D* shows the original data and the fitting result with these two models.

These rather simplified models have several limitations. First, these models do not consider the effect of stromal component of tumors including tumor blood vessels, extracellular matrix and stromal cells. Second, the distribution of necrotic tissues can be heterogeneous with different tumors. However, our tumor samples based on H&E staining shows a marked central tumor necrosis with viable tumor rim at the tumor edges with comparable thickness as estimated by Model 2 (Fig. 3*C*). Thus, this analysis supports that the differences in caliper-based tumor volume and the blood Gluc measurements are largely due to the presence of non-viable necrotic tissues.
